# Multimodality treatment in immunocompromised patients with severe COVID‐19: the role of IL‐6 inhibitor, intravenous immunoglobulin, and haemoperfusion

**DOI:** 10.1002/rcr2.733

**Published:** 2021-03-07

**Authors:** Nophol Leelayuwatanakul, Napplika Kongpolprom, Thitiwat Sriprasart, Vorakamol Phoophiboon, Vorawut Thanthitaweewat, Sarita Thawanaphong, Worawan Sirichana, Naricha Chirakalwasan, Kamon Kawkitinarong, Chanchai Sittipunt, Opass Putcharoen, Leilani Paitoonpong, Gompol Suwanpimolkul, Watsamon Jantarabenjakul, Nattachai Srisawat, Monvasi Pachinburavan

**Affiliations:** ^1^ Division of Pulmonary and Critical Care Medicine, Department of Medicine, Faculty of Medicine Chulalongkorn University Bangkok Thailand; ^2^ Division of Infectious Diseases, Department of Medicine, Faculty of Medicine Chulalongkorn University Bangkok Thailand; ^3^ Thai Red Cross Emerging Infectious Diseases Clinical Center King Chulalongkorn Memorial Hospital Bangkok Thailand; ^4^ Department of Paediatrics, Faculty of Medicine Chulalongkorn University Bangkok Thailand; ^5^ Division of Nephrology, Department of Medicine, Faculty of Medicine Chulalongkorn University Bangkok Thailand

**Keywords:** COVD‐19, haemoperfusion, IVIG, SARS‐CoV‐2, tocilizumab

## Abstract

Cytokine release syndrome (CRS) is known to be associated with severe coronavirus disease 2019 (COVID‐19). Multiple anti‐inflammatory therapies such as tocilizumab, corticosteroids, intravenous immunoglobulin (IVIG), and haemoadsorption or haemoperfusion have been used to combat this life‐threatening condition. However, immunocompromised hosts are often omitted from research studies, and knowledge on the clinical efficacy of these therapies in immunocompromised patients is therefore limited. We report two cases of immunocompromised patients with severe COVID‐19‐related CRS requiring mechanical ventilation who were treated with multimodality treatment consisting of tocilizumab, IVIG, and haemoperfusion. Within 48 h, both patients showed clinical improvement with PaO_2_:FiO_2_ ratio and haemodynamic stability. Both survived to discharge. There were no adverse events following these therapies. In conclusion, combined therapeutic modalities, possibly tailored to individual inflammatory profiles, are promising treatment for severe COVID‐19 infection in the immunocompromised host. Timely administration of adjunctive therapies that alleviate overwhelming inflammation may provide the best outcome.

## Graphical abstract

We report two cases of immunocompromised patients with severe coronavirus disease 2019 (COVID‐19)‐related cytokine release syndrome requiring mechanical ventilation who were treated with multimodality treatment consisting of tocilizumab, intravenous immunoglobulin (IVIG), and haemoperfusion. Within 48 h, both patients showed clinical improvement with PaO_2_:FiO_2_ ratio and haemodynamic stability. Both survived to discharge. There were no adverse events following these therapies.

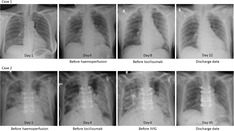

## Introduction

In December 2019, a cluster of unexplained pneumonia cases were initially diagnosed in Wuhan, the capital of Hubei province, China. A new beta‐coronavirus, now named severe acute respiratory syndrome coronavirus 2 (SARS‐CoV‐2), was identified as the cause of coronavirus disease 2019 (COVID‐19). Two months after the index case of COVID‐19 pneumonia in Wuhan, the disease outbreak of COVID‐19 acute respiratory disease resulted in more than 70,000 confirmed cases and 2718 deaths officially reported in mainland China. On 30 January 2020, WHO declared a Public Health Emergency of International Concern regarding the outbreak of COVID‐19 in China. The pandemic has since infected more than 90 million people resulting in over 2 million deaths worldwide.

Among COVID‐19 patients, clinical symptoms may range from mild upper respiratory tract symptoms to severe acute respiratory distress syndrome (ARDS), with some progressing to multiorgan failure [1]. Initial retrospective studies in China reported that patients requiring intensive care unit (ICU) admission had elevated inflammatory cytokines and chemokines in serum compared to those not requiring ICU admission which suggest that cytokine release syndrome (CRS) might play an important role in the pathogenesis of severe COVID‐19 infection [2]. Furthermore, interleukin (IL)‐6 was also found to be elevated in severe COVID‐19 infection which is suspected to be driven by viral infection; therefore, IL‐6 receptor blockade may potentially be an effective treatment for this resulting overwhelming inflammation. Treatment for COVID‐19 pneumonia has been focused on both eradicating the virus itself as well as diminishing the inflammatory response from cytokine storms, which is believed to be responsible for multiorgan failure in patients infected with COVID‐19.

While established protocols for patients with severe disease are currently available, immunocompromised patients are often omitted from clinical studies. As a result, the efficacy of adjunctive treatment in immunocompromised patients is commonly extrapolated from studies in normal hosts. Clinical experience in immunocompromised hosts is limited to case reports and case series. In addition to standard treatment, intravenous immunoglobulin (IVIG), haemoadsorption, and tocilizumab would be potential therapies in those with severe disease and may be even more beneficial in patients who are immunocompromised.

We report two immunocompromised patients with severe COVID‐19 pneumonia who successfully underwent multimodality treatment with tocilizumab, IVIG, and haemoperfusion as adjunctive therapies. Evidence on anti‐inflammatory therapies as adjunctive treatment for severe COVID‐19‐related CRS is also reviewed.

## Case Report

**Figure 1:**
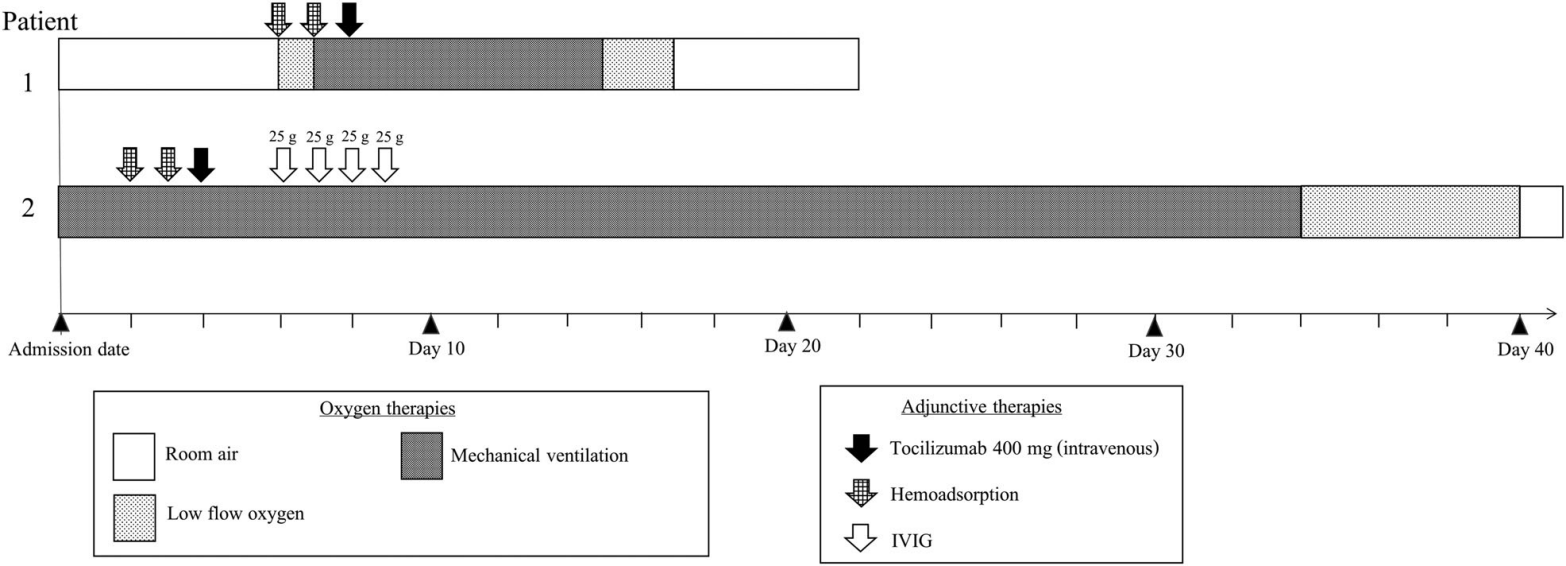
Summary of mode of oxygen therapy and adjunctive treatments given in each patient in time sequence.

**Figure 2:**
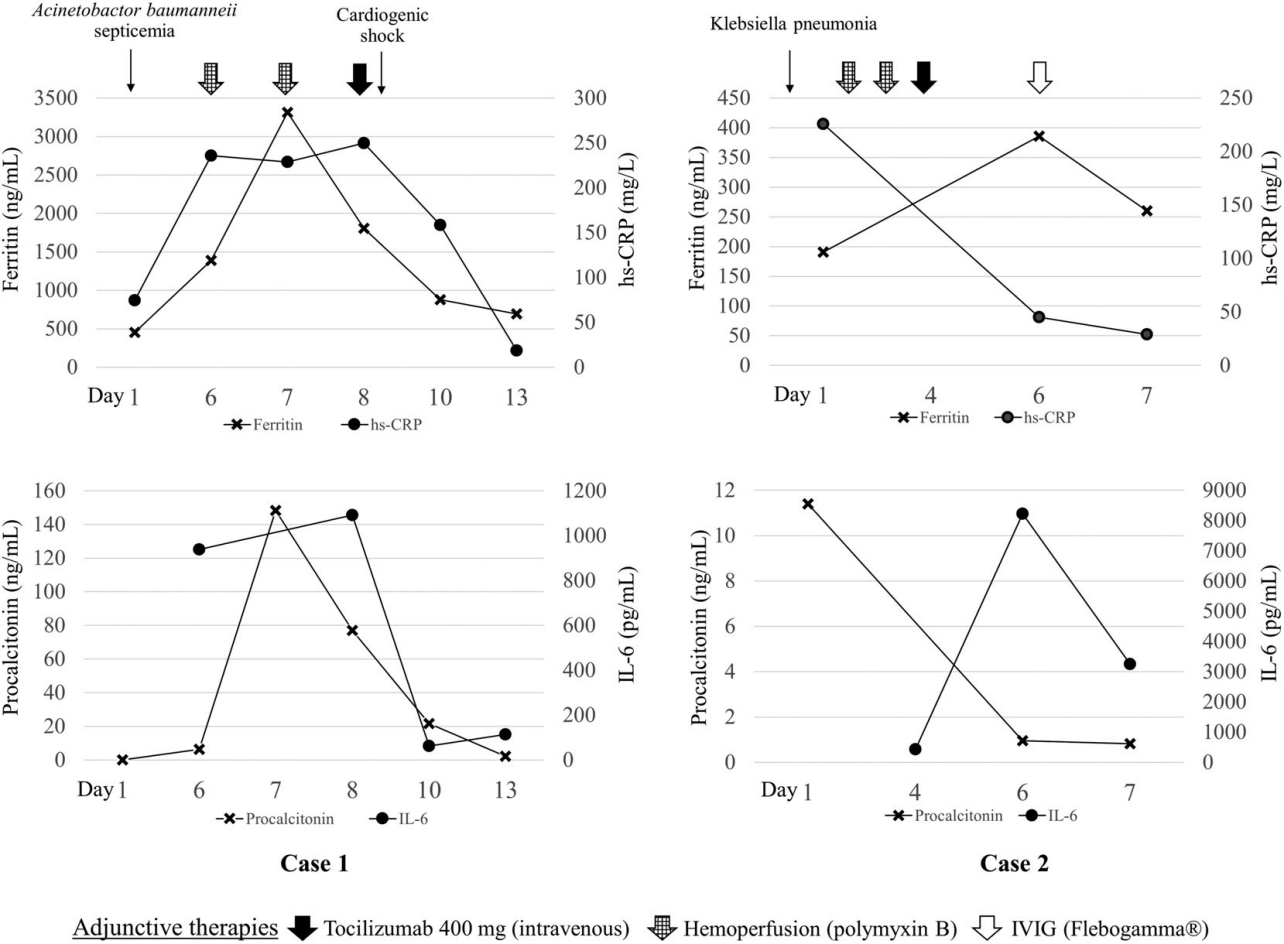
Laboratory investigations of each patient in time sequence.

We, herein, reported two patients with severe COVID‐19 pneumonia who were treated with favipiravir‐based regimen combined with other antimicrobial agents. Multimodality treatment with tocilizumab, IVIG, and haemoperfusion was used as adjunctive therapy. Both patients were on mechanical ventilation using lung protective ventilation strategies, sedation, and neuromuscular blocking agent. Mode of oxygen therapy, laboratory data, and adjunctive treatments given in each patient were summarized in time sequence as shown in Figures 1 and 2.

### Case 1

**Figure 3:**
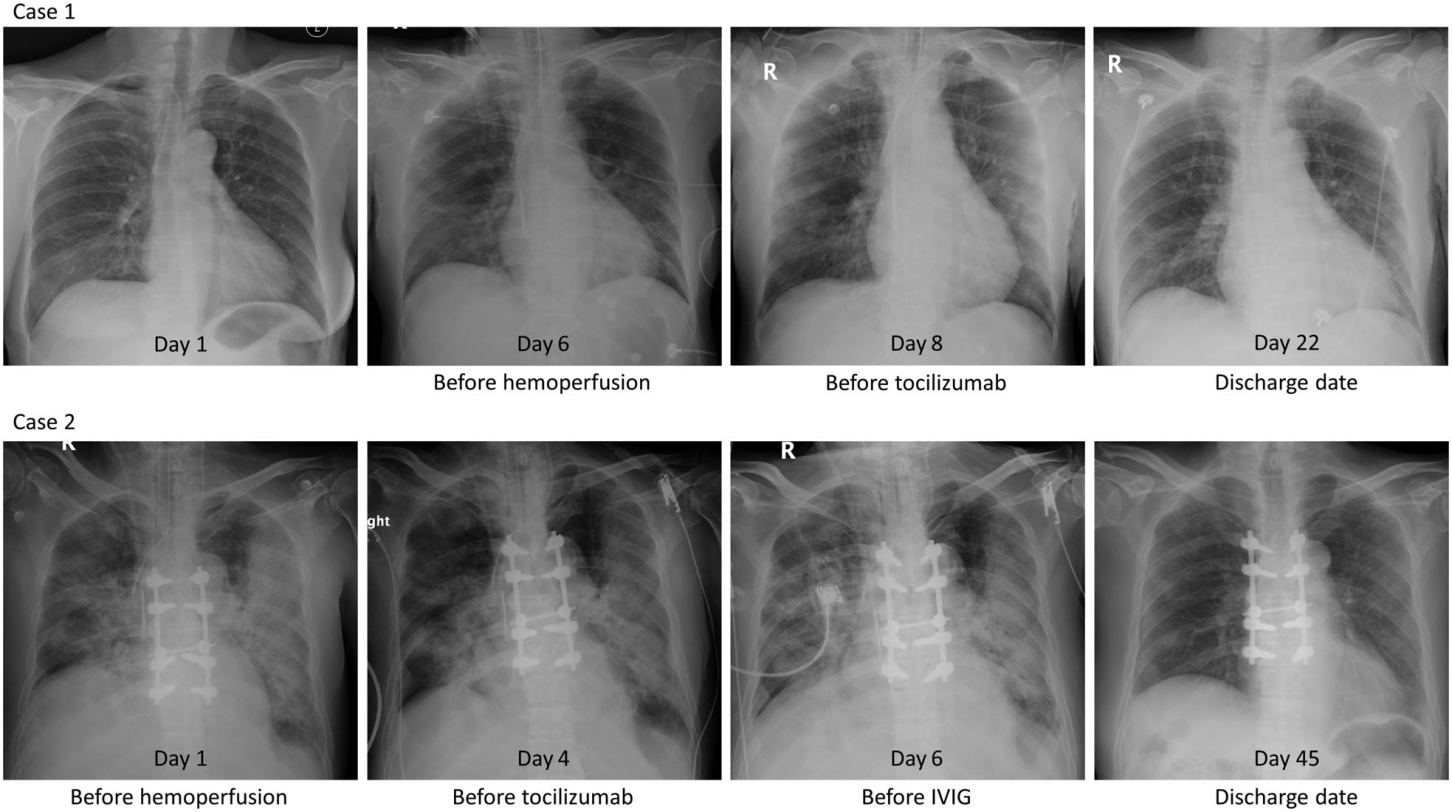
Chest radiographs on initial presentation, on the day before each adjunctive therapy, and on discharge date.

A 58‐year‐old woman with a known history of HIV infection, diabetes, and dyslipidaemia was presented. Her last CD4 count was 700 cell/mm^3^ with good virological suppression. She had fever, cough, and myalgia for six days prior to admission. The real‐time reverse transcriptase‐polymerase chain reaction (RT‐PCR) from her nasopharyngeal and throat swab was positive for SARS‐CoV‐2. No pulmonary opacity was shown in her initial chest X‐ray (Fig. 3). Favipiravir, hydroxychloroquine (HCQ), and darunavir/ritonavir (DRV/r) were administered.

On day 6 of admission, she developed dyspnoea with SpO_2_ (peripheral capillary oxygen saturation, on ambient air) of 93%. Her follow‐up chest X‐ray showed multifocal patchy opacity in both middle and lower lung zones. Haemoculture was identified as *Acinetobactor baumannii*. Her clinical condition rapidly progressed to ARDS on the same day which required invasive mechanical ventilation (PaO_2_:FiO_2_ ratio was 260). Repeat RT‐PCR for SARS‐CoV‐2 was still positive with cycle threshold (ct) of 20. COVID‐19‐induced CRS and co‐infection with *A*. *baumannii* septicaemia were thought to be responsible for clinical deterioration. Two sessions of polymyxin B haemoperfusion (PMX‐HP) were performed on days 6 and 7 of admission. Ampicillin‐sulbactam, colistin, and sulperazone were also given empirically which later de‐escalated to ceftazidime.

After the second session of haemoperfusion, her body temperature returned to normal but became hypotensive. Echocardiography revealed a cardiac ejection fraction of 30% and apical wall hypokinesia consistent with stress‐induced cardiomyopathy. The patient was started on norepinephrine and dobutamine for combination septic and cardiogenic shock. Inflammatory markers were elevated: IL‐6 was 1091 pg/mL, ferritin was 3316 ng/mL, hs‐CRP was 228 mg/L, D‐dimer was 2174 ng/mL, and lactate dehydrogenase (LDH) was 492 U/L. Her chest X‐ray showed progression to diffuse bilateral ground‐glass and patchy opacity (Fig. 3). A single dose of tocilizumab (400 mg) was administered intravenously. Her clinical status was improved significantly after haemoperfusion followed by tocilizumab. Vasopressors were weaned off and PaO_2_:FiO_2_ ratio was increased to 500 within 48 h of tocilizumab administration. She remained on invasive mechanical ventilation for another five days and was successfully extubated on day 12 of admission. She was discharged from the hospital with a total length of stay of 22 days. The time to negative RT‐PCR for SARS‐CoV‐2 was 15 days after admission.

### Case 2

A 69‐year‐old man, partially dependent on most activity of daily living, with a known history of relapse multiple myeloma (MM) IgG kappa stage II presented with spinal cord compression at the level of T3–T7 receiving laminectomy and fusion surgery, triple vessel disease, diabetes, and hypertension. He presented at emergency room with dyspnoea and myalgia for one week. His vital signs were body temperature (BT) 37.4°C, respiratory rate (RR) 30/min, blood pressure 60/40 mmHg, and SpO_2_ (on ambient air) 64%. On admission (seventh day of symptom onset), he was mechanically ventilated due to hypoxaemic respiratory failure. The RT‐PCR for SARS‐CoV‐2 was detected from nasopharyngeal swab and tracheal suction. The initial chest X‐ray showed bilateral patchy opacity (Fig. 3). Favipiravir, HCQ, azithromycin, and ceftriaxone were administered afterwards. His haemodynamic stability was maintained by norepinephrine. Fludrocortisone was concomitantly administered with a high suspicion of autonomic dysreflexia as the history of spinal cord compression.

On day 2 of admission, due to worsening of his haemodynamic stability and oxygenation (PaO_2_:FiO_2_ ratio of 130), we decided to initiate PMX‐HP for two consecutive days (days 2 and 3 of admission). The level of endotoxin activity assay (EAA) before haemoperfusion was 0.58 U. On day 4 (11th day of symptom onset), he still had haemodynamic instability and worsening hypoxaemia requiring higher dose of norepinephrine and FiO_2_ (PaO_2_:FiO_2_ ratio of 143). Laboratory results showed elevation of the inflammatory markers: IL‐6 was 426.2 pg/mL, ferritin was 190.5 ng/mL, hs‐CRP was 225 mg/L, D‐dimer was 7979 ng/mL, and LDH was 321 U/L. The sputum culture was identified as *Klebsiella pneumoniae*. Single dose of tocilizumab (400 mg) and piperacillin‐tazobactam was administered. Nevertheless, the patient's condition did not improve including haemodynamic instability and persistent low PaO_2_:FiO_2_ ratio; 25 mg of IVIG (Flebogamma®, Grifols, DKSH, Spain) was administered for four consecutive days (days 6–9 of admission). Eventually, the PaO_2_:FiO_2_ ratio was increased to 180–230 and norepinephrine could be weaned off on day 13. His clinical condition was complicated with secondary bacterial cellulitis which was managed by antibiotic. However, he could be successfully extubated with a total 31 days of mechanical ventilation and discharged with a total length of stay of 45 days. The time to negative RT‐PCR for SARS‐CoV‐2 was 21 days.

## Discussion

CRS‐related ARDS and secondary haemophagocytic lymphohistiocytosis (sHLH) are two distinct constellation of symptoms which are believed to attribute to the high mortality rate among COVID‐19 patients with respiratory failure [3, 4, 5]. CRS has previously been observed following chimeric antigen receptor (CAR) T‐cell therapy and therapeutic antibodies administration but has also been seen in certain viral infections such as SARS‐CoV‐1 and now in SARS‐CoV‐2. In addition, the characteristics of hyperinflammation reported in severe COVID‐19 infection also resemble sHLH triggered by other viral infections [2]. However, the clinical syndrome of these two entities might be indistinguishable. Early detection of hyperinflammatory state and selection of life‐saving therapies should be done to prevent clinical deterioration in severe COVID‐19 patients.

In our critical cases of COVID‐19, both were immunocompromised patients with hypoxaemic respiratory failure requiring invasive mechanical ventilation. At the time of the first wave of the pandemic in Thailand, there were limited clinical data available for the treatment of severe COVID‐19. Aiming to prevent clinical deterioration in patients with CRS, tocilizumab, IVIG, haemoperfusion, and corticosteroids had been introduced as part of treatment in our centre. Dhakal et al. reported the overall mortality rate of 57% from the case series of MM patients infected with COVID‐19 and none of them received tocilizumab [6]. Up to now, additional data on the clinical efficacy of these therapeutic modalities used for coronaviruses were published, particularly COVID‐19 infection. These adjunctive treatments will be discussed and reviewed.

### Tocilizumab

The upregulation of cytokines and chemokines, IL‐6, is thought to be a major step in the immunopathogenesis of COVID‐19‐related ARDS. Inhibition of IL‐6 should therefore theoretically be effective in suppressing overwhelming inflammation found in COVID‐19 [7]. Tocilizumab is a humanized anti‐IL‐6 receptor which is Food and Drug Administration (FDA)‐approved for use in rheumatoid arthritis, CAR T‐cell‐induced CRS, and various other rheumatological conditions. Off‐label use of tocilizumab in severe COVID‐19 infection was found to be effective in the treatment of COVID‐19 patients [8].

In critically ill patients infected with COVID‐19, remarkable improvement of respiratory parameters and return of body temperature to normal were observed after tocilizumab treatment [9, 10, 11]. Another observational study of combination of low‐dose methylprednisolone and tocilizumab was conducted in 15 patients at a single centre in China. Patients who achieved normalization of IL‐6 levels with tocilizumab and methylprednisolone had clinical stabilizations rather than disease progression [12]. In contrast, some retrospective case–control study failed to demonstrate the mortality benefit of using tocilizumab in severe COVID‐19 patients [13, 14]. Of note, another non‐randomized observational study using intravenous or subcutaneous tocilizumab in severe or critical COVID‐19 patients showed significantly higher survival rates compared to patients not receiving tocilizumab [15, 16]. Recently, the preliminary result of randomized controlled COVACTA trial and BACC bay trial showed disappointing result of tocilizumab that failed to improve the patient mortality or intubation in moderately severe non‐intubated COVID‐19 [17, 18]. However, a lower primary outcome event rate of intubation and death may have limited the treatment effect of tocilizumab in this study. In contrast, sarilumab, another IL‐6 inhibitor, did show a trend towards decreased mortality in a recent randomized controlled study in critically ill patients [19]. It remains to be seen whether IL‐6 inhibitors may be of use in our most critical patients.

In addition, in a case of extremely high level of IL‐6, repeated dose of tocilizumab is suggested [12]. The prospective study in Italy also reported that two to three doses of tocilizumab (800 mg), 12–24 h apart, may be an effective treatment in COVID‐19 pneumonia‐related hyperinflammatory syndrome [11]. However, as our immunocompromised cases with worsening CRS despite receiving single dose of tocilizumab, the IL‐6 level in the second case was still high (Fig. 2). Given the risk of its immunomodulatory effect, we believed that the risks of repeated dose might outweigh benefits in our cases. Furthermore, the clinical efficacy of tocilizumab in immunocompromised patients was reported in one case of MM patient who was successfully treated with tocilizumab as well as a case series in kidney transplant recipients [20, 21]. Other than case reports and series, there are no controlled trials on the use of IL‐6 inhibitors in immunocompromised hosts with COVID‐19. Hence, the risk–benefit profile of using tocilizumab in such cases should be carefully considered.

### Intravenous immunoglobulin

Data on the immunomodulatory effects of IVIG have been shown to regulate multiple steps in complex network of immune systems depending on the immunopathogenesis of those diseases. Fc receptor‐mediated effect, regulation of T cells, regulation of B cells and antibodies, activation of complements, cytokines neutralization and inhibition of downstream mediator gene transcription, toxin scavenging, and regulation of apoptosis were demonstrated to be the responsible immunomodulatory pathways [22, 23, 24]. However, immunoglobulin was prepared from the plasma of a donor who had high titres of neutralizing activities against a specific organism, so‐called hyperimmune IVIG (H‐IVIG). Both IVIG and H‐IVIG have always been introduced to be part of the treatment regimen or prevention of the emerging infectious diseases.

The efficacy of IVIG or H‐IVIG in viral infection had been investigated and found to be of potential benefit in specified viral subgroups. During the pandemic of 2009 influenza A(H1N1), the early use of H‐IVIG within five days of symptom onset was associated with lower viral load. However, the survival benefit of H‐IVIG with neutralizing activities against H1N1 strain over normal IVIG without its neutralizing activities remains unclear [25]. Furthermore, H‐IVIG using high‐titre anti‐influenza plasma also failed to show survival benefit in treating hospitalized patients infected with influenza A or B compared to placebo [26].

The published studies of IVIG in treating SARS‐CoV infection were also inconclusive due to the retrospective nature of the study. IVIG may be effective in selected SARS‐CoV patients with leukopenia and/or thrombocytopenia, radiological progression of the chest radiography, or haemophagocytic syndrome [27, 28]. However, none of the studies failed to demonstrate its efficacy over other therapy, such as ribavirin and corticosteroids. There is also lack of evidence to prove the efficacy of IVIG in Middle East respiratory syndrome coronavirus (MERS‐CoV) patients. Experimental studies on animal model of human polyclonal immunoglobulin (G) antibodies, derived from transchromosomic bovines, demonstrated high neutralizing antibody titres in vitro and reduced MERS‐CoV titres in animal model [29]. Some retrospective studies reported the clinical outcome of IVIG in a subgroup of patients, but a large clinical trial is still required to demonstrate its efficacy [30]. Recently published studies of currently marketed IVIG (Gamunex®‐C, Grifols, DKSH, Spain and Flebogamma dual inactivation and filtration (DIF)) were demonstrated to have positive reactivity against MERS‐CoV, SARS‐CoV, and SARS‐CoV‐2 in vitro [31], although the effect of enhancing viral clearance in vivo is still lacking.

On the other hand, the efficacy of IVIG is not limited to those neutralizing activities but is also used to ameliorate the immune response in CRS. As for evidence in MERS‐CoV and SARS‐CoV, IVIG is another promising therapy exhibiting clinical benefits in selected patient with clinically suspected CRS [27, 32]. There is substantial evidence to prove the efficacy of polyvalent IVIG in COVID‐19 patients. The clinical outcomes of critically ill COVID‐19 patients who received IVIG as part of the therapy were reported in the large cohort study, so its efficacy could not be concluded [33]. However, one retrospective study analysed that the clinical outcomes of IVIG in severe COVID‐19 pneumonia as an adjunctive therapy initiated within 48 h of ICU admission could reduce the use of mechanical ventilation, length of stay in hospital and ICU, and also 28‐day mortality [34]. A recent observational report of COVID‐19 patients, given five days of high dose of IVIG (0.3–0.5 g/kg/day), had also been shown to be effective in preventing clinical deterioration and intubation [35, 36]. Moreover, a multicentre retrospective cohort study in China also could not address the overall survival benefit of IVIG but found that, in critical patients who use high dose IVIG (>15 g/day) it could significantly reduce 28‐ and 60‐day mortality (*P* = 0.002 and *P* < 0.0001, respectively). Early initiation of IVIG within seven days of admission could also reduce 60‐day mortality, total in‐hospital stay, and total course of disease, and increase survival time [37].

To the best of our knowledge, polyvalent IVIG is another rescue therapy in COVID‐19‐related CRS regarding its anti‐inflammatory effects as mentioned above. In our cases, IVIG was also prescribed as a part of adjunctive therapies. However, appropriate timing, doses, and preparation of IVIG remain unclear and require well‐designed study to support their benefits. Therefore, administration of IVIG should be considered only in severe COVID‐19 with CRS as its efficacy has not been well elicited from previous study. Convalescent plasma or H‐IVIG may be an attractive additional treatment in severe COVID‐19 infection. Nevertheless, protocols for collection, preparation, and administration in real‐world practice are diverse and extremely challenging.

### Haemoperfusion

There are limited data on the use of haemoadsorption for the treatment of COVID 19 specifically. However, several extracorporeal blood purification systems (e.g. CytoSorb, CytoSorbents Corporation, Monmouth Junction, NJ, USA and oXiris, GAMBRO Industries, France) that are designed theoretically for adsorption of endogenous pathogenic mediators and pro‐inflammatory cytokines have been temporarily approved by the FDA under Emergency Use Authorization (EUA) programme for compassionate use in patients with severe COVID‐19 infection.

In our cases, PMX‐HP was used based on the high level of EAA, of which intermediate to high level (more than 0.4 U) could demonstrate endotoxaemia and be associated with worse clinical outcomes [38]. Concomitant bacterial infections were reported as described previously and might be related with high EAA level in our cases. Sirivongrangson et al. reported endotoxaemia and circulating bacterial DNA in COVID‐19 pneumonia that was proposed to result from secondary bacterial translocation, which could contribute to cytokine storm and multiorgan failure [39]. The clinical efficacy of PMX‐HP had been demonstrated by case series from EUPHAS2 registry which was associated with haemodynamic improvement and organ function recovery in COVID‐19 patients with a history of septic shock from secondary bacterial infection [40]. Moreover, Ishiwari et al. also reported a successful treatment of PMX‐HP in a case of COVID‐19‐associated hypercytokinaemia‐induced severe respiratory failure who had been treated with methylprednisolone (1 g) for three days [41]. However, the clinical benefit of using haemoadsorption and haemoperfusion in COVID‐19 patients and pilot studies remains debatable. As presented here, the haemoperfusion combined with other life‐saving therapies was shown to be effective in COVID‐19‐related CRS. Therefore, we believe that using these extracorporeal therapies are of potential theoretical benefit and should be reserved in severe cases that do not respond to standard‐of‐care treatment, particularly dexamethasone.

To summarize, several novel adjunctive therapies have been shown to mediate the inflammatory response in critically ill patients. Based on previous data and clinical experience with other coronaviruses, IVIG, convalescent plasma, corticosteroids, and tocilizumab are potential treatments to combat the unusually aggressive inflammatory response that is mostly associated with detrimental outcomes for COVID‐19 patients. As our patients demonstrated, multimodality treatments may be effective in critical COVID‐19 patients. We demonstrate here that IL‐6 inhibitors, IVIG, and haemoadsorption can be used safely in selected immunocompromised hosts. Many confounding factors such as severity of disease, co‐morbid conditions, and co‐infections may potentially obfuscate the effects of tocilizumab in combination with other adjuvant treatments. However, we do believe that the multiple pathways of the pathogenesis of COVID‐19 should warrant multimodality treatment rather than a single agent inhibiting one single pathway. Using multiple adjuvant treatments attacking the various mechanisms of inflammation may potentially lead to better outcomes for our most critical patients.

In conclusion, we suggest that timely administration of adjunctive therapies aiming to alleviate inflammation in critically ill COVID‐19‐infected patients, whose laboratory parameters suggest severe hyperinflammation, may influence the mortality outcome. No single therapy has been proven to defeat CRS in its entirety. Combined therapeutic modalities, possibly tailored to individual inflammatory profiles, should be considered in these complex patients and may produce better outcomes, as shown here.

### Disclosure Statements

Appropriate written informed consent was obtained for publication of this case report and accompanying images.

This study was approved by the Faculty of Medicine, Chulalongkorn University (IRB 348/63).

At the time this report was accepted for publication, the authors declared that the patients in this report had not been included in any previously published report on COVID‐19 that they had authored.

### Author Contribution Statement

Conceptualization: Nophol Leelayuwatanakul, Monvasi Pachinburavan. Methodology: Nophol Leelayuwatanakul, Monvasi Pachinburavan. Formal analysis: Nophol Leelayuwatanakul, Vorawut Thanthitaweewat, Sarita Thawanaphong, Watsamon Jantarabenjakul. Investigation: Nophol Leelayuwatanakul, Vorawut Thanthitaweewat, Sarita Thawanaphong, Watsamon Jantarabenjakul. Resources: Nophol Leelayuwatanakul, Napplika Kongpolprom, Thitiwat Sriprasart, Vorakamol Phoophiboon, Vorawut Thanthitaweewat, Sarita Thawanaphong, Worawan Sirichana, Naricha Chirakalwasan, Kamon Kawkitinarong, Chanchai Sittipunt, Opass Putcharoen, Leilani Paitoonpong, Gompol Suwanpimolkul, Watsamon Jantarabenjakul, Nattachai Srisawat, Monvasi Pachinburavan. Data curation: Nophol Leelayuwatanakul, Vorawut Thanthitaweewat, Sarita Thawanaphong, Watsamon Jantarabenjakul. Writing—original draft preparation: Nophol Leelayuwatanakul. Writing—review and editing: Monvasi Pachinburavan. Visualization: Napplika Kongpolprom, Thitiwat Sriprasart, Vorakamol Phoophiboon, Vorawut Thanthitaweewat, Sarita Thawanaphong, Worawan Sirichana, Naricha Chirakalwasan, Kamon Kawkitinarong, Chanchai Sittipunt, Opass Putcharoen, Leilani Paitoonpong, Gompol Suwanpimolkul, Watsamon Jantarabenjakul, Nattachai Srisawat, Supervision: Monvasi Pachinburavan. Project administration: Nophol Leelayuwatanakul, Monvasi Pachinburavan. All authors have read and agreed to the published version of the manuscript.
